# Neonatal transitional support with intact umbilical cord in assisted vaginal deliveries: a quality-improvement cohort study

**DOI:** 10.1186/s12884-020-03188-0

**Published:** 2020-08-27

**Authors:** Elisabeth Sæther, Friedrich Reinhart-Van Gülpen, Christer Jensen, Tor Åge Myklebust, Beate Horsberg Eriksen

**Affiliations:** 1 Department of Obstetrics and Gynecology, Møre and Romsdal Hospital Trust, Åsehaugen 5, N-6017 Ålesund, Norway; 2 Department of Pediatrics, Møre and Romsdal Hospital Trust, Ålesund Hospital, Ålesund, Norway; 3 Department of Medicine and Healthcare, Møre and Romsdal Hospital Trust, Ålesund Hospital, Ålesund, Norway; 4 Department of Health Sciences in Ålesund, Faculty of Medicine and Health Sciences, Norwegian University of Science and Technology (NTNU), Ålesund, Norway; 5 Department of Research and Innovation, Helse Møre and Romsdal Hospital Trust, Ålesund, Norway

**Keywords:** Umbilical cord clamping, Infant, Resuscitation, Assisted vaginal delivery

## Abstract

**Background:**

Deferring cord clamping has proven benefits for both term and preterm infants, and recent studies have demonstrated better cardio-respiratory stability if clamping is based on the infant’s physiology, and whether the infant has breathed. Nevertheless, current guidelines for neonatal resuscitation still recommend early cord clamping (ECC) for compromised babies, unless equipment and competent personnel to resuscitate the baby are available at the mother’s bedside. The objective of this quality improvement cohort study was to evaluate whether implementing a new delivery room protocol involving mobile resuscitation equipment (LifeStart™) reduced the prevalence of ECC in assisted vaginal deliveries.

**Methods:**

Data on cord clamping and transitional care were collected 8 months before and 8 months after implementing the new protocol. The Model for Improvement was applied to identify drivers and obstacles to practice change. Statistical Process Control analysis was used to demonstrate signals of improvement, and whether these changes were sustainable. Multivariate logistic regression was used to evaluate the impact of the new protocol on the primary outcome, adjusted for possible confounders.

**Results:**

Overall prevalence of ECC dropped from 13 to 1% (*P* < 0.01), with a 98% relative risk reduction for infants needing transitional support on a resuscitation table (adjusted OR 0.02, *P* < 0.001). Mean cord clamping time increased by 43% (*p* < 0.001). Although fewer infants were placed directly on mothers’ chest (*n* = 43 [42%] vs *n* = 69 [75.0%], *P* < 0.001), there were no significant differences in needs for immediate transitional care or transfers to Neonatal Intensive Care Unit. A pattern of improvement was seen already before the intervention, especially after mandatory educational sessions and cross-professional simulation training.

**Conclusions:**

A new delivery-room protocol involving mobile resuscitation equipment successfully eliminated early cord clamping in assisted vaginal deliveries of term and near-term infants. A systematic approach, like the Model for Improvement, seemed crucial for both achieving and sustaining the desired results.

**Trial registration:**

The study was approved as a service evaluation as defined by the Regional Committee for Medical and Health Research Ethics (2018/1755/REK midt).

## Background

Optimal timing of umbilical cord clamping has been debated for centuries, and definitions vary [1]. “Early” cord clamping (ECC) is generally done before one minute after birth (commonly 15–30 seconds), whereas “delayed” cord clamping (DCC) refers to later than 1 minute, or when cord pulsations have ceased [2]. Historically, doctors and midwives have defined ECC as malpractice, associated with risk and harm for both mother and infant [3].

In Norway, DCC was the norm in midwife-lead deliveries until late 1990s. In many hospitals, a shift followed the introduction of STAN™ technology for continuous foetal heart rate monitoring. The user manual recommended immediate “double-clamping” of the umbilical cord before the baby’s first breath (ICC) to obtain correct cord blood samples for blood gas analysis [4]. Quickly, cord blood analysis became a quality benchmark, recommended for all deliveries [5]. Despite evidence supporting sampling from a pulsating cord [6], many institutions still practice ICC, especially in assisted vaginal deliveries (ventouse-, forceps- or breech) and caesarean sections [7].

A growing body of evidence calls for abandoning ECC [8, 9]. The World Health Organization (WHO) recommends DCC (at least 1 min), unless concern about mother or baby [10]. ECC is recommended if the baby needs to be moved for resuscitation. However, cardio-respiratory support may be initiated with an intact umbilical cord, provided that competent personnel and equipment are available at the mother’s bedside [10]. This corresponds with international guidelines on neonatal resuscitation and transitional care [11, 12]. When DCC is not feasible, umbilical cord milking (UCM) has been suggested as a safe alternative for infants with gestational age 29 weeks or more [13, 14].

Research on infant transitional physiology underpins the importance of maintaining vital parameters like blood pressure, organ perfusion and oxygen saturation during the first minutes of life [15, 16]. This urges a new look on how babies at risk are handled in the delivery rooms [17]. Experts argue that these infants would be better off if respiratory support were provided with the umbilical cord intact [18]. Immediate cord clamping is a non-physiological intervention that blocks venous return from the placenta to the baby’s heart and obstructs umbilical arteries, thus reducing preload and increasing afterload and peripheral vascular resistance, resulting in reduced cardiac output [19].

New research shows that both venous and arterial umbilical flow is unrelated to cessation of pulsations, with large individual variations [20]. Researchers argue that cord clamping should be based on the infants’ physiology rather than a fixed period of time [15]. Randomized controlled trials (RCT) suggest benefits of waiting longer than 2–3 min. For term infants, studies have found increased iron stores and brain myelinisation [21], better oxygenation and perfusion of vital organs with DCC [22].

Most trials comparing cord clamping practices exclude complicated vaginal deliveries and babies needing resuscitation, whereby conclusions cannot be drawn for these situations [10, 23]. Assisted vaginal deliveries are often complicated by intrapartum events like foetal asphyxia, umbilical cord compression or shoulder dystocia, frequently delivering compromised and hypovolemic babies. To move the baby for ventilation support or resuscitation, the umbilical cord must be cut; although the essential needs are volume and oxygen, both readily available via the placenta and umbilical cord [24, 25].

However, ventilation support and full resuscitation can be done without separating mother and baby [22, 26]. For hospital settings, mobile bed-side resuscitation equipment (ex. LifeStart™, Concord™) has been developed and tested in different settings with positive results, and is welcomed by both parents and health care professionals [27–29]. Early skin-to-skin-contact and the familiar voice and touch of the mother is shown to improve transition and reduce stress, even when transitional support is needed [30]. Experts argue that separation of infants from their mothers at birth is no longer acceptable [31], and that delivery rooms should be furnished to secure optimal contact between mothers and infants [32].

The Medical Birth Registry of Norway does not require documentation of cord clamping time in birth-records. Therefore, institution statistics on cord clamping practices are not available. After implementation of an evidence-based guideline for DCC at our specialised (level one) obstetric unit, compliance was tested by measuring the time from delivery to cord clamping in all deliveries during March, 2017. A Patient-Safety and Quality Improvement Project was introduced, aiming to ensure optimal cord clamping for all infants.

Consequently, as a continuation of the quality improvement (QI) initiative and with support from the above mentioned evidence, the objective of this study was to evaluate whether implementing a new delivery-room protocol involving a mobile resuscitation trolley (LifeStart™) reduced the prevalence of ECC in assisted vaginal deliveries.

## Methods

### Study design

We conducted a quality improvement cohort QI study of cord clamping practice in assisted vaginal deliveries. The study consisted of two phases: A baseline 8-month period (Period 1), where standard care was applied (March 1st to October 31st 2017), followed by an 8-month intervention-period (Period 2), where the new protocol and resuscitation equipment were implemented (November 1st 2017 to June 30th 2018). We used anonymous data from the previously described Patient Safety and Quality Improvement Project. The study was approved as a service evaluation as defined by the Regional Committee for Medical and Health Research Ethics (2018/1755/REK midt), and waiver of individual patient informed consent was granted. It was approved by the Director of Clinic for Women, Children and Adolescents, Møre and Romsdal Hospital Trust, and by the institution’s Privacy Ombudsman (ephorte reference 2018/1357–10).

### Setting

The study was performed at Clinic for Women, Children and Adolescents, Møre and Romsdal Hospital Trust, Ålesund, Norway. At this clinic, low-risk deliveries are midwife-led, whereas complicated deliveries are led by teams consisting of an obstetrician, an obstetric registrar and a midwife. A Neonatal Intensive Care Unit (NICU) facilitates care for sick and premature infants. Whenever possible, the collaborative conduct of assisted vaginal deliveries is planned prior to delivery with a paediatric team (paediatric registrar and neonatal intensive care nurse), expected to be present in the delivery room and responsible for initial assessment and treatment of the infant. An evidence based protocol of delayed cord clamping was introduced in 2009.

### Standard care (Period 1)

Risk assessments and differentiation of care were guided by national clinical standards, and done during pregnancy, at patient admission and during labour. Preparations in the delivery room were done accordingly. Paediatric team was alarmed when breech birth was imminent, or decision of instrumentation was made. A resuscitation table (Cosy-Cot™, Fisher & Paykel Healthcare, Auckland, NZ) was brought into the room and placed opposite to the birthing bed.

Delayed cord clamping was attempted in all deliveries, while providing essential care. An assistant midwife obtained arterial and venous cord blood gases from a pulsating cord within the first 30–40 s. Apgar Scores were evaluated after 1, 5 and 10 min by the paediatric team. Neonatal resuscitation algorithm was applied [33].

If the infant was compromised and not recovering during the first 30–60 s, the umbilical cord was cut at least 30 cm from the umbilicus, and the infant was moved to the resuscitation table for further assessment and care. Cord clamping time was recorded by an assistant nurse, using a digital clock in the delivery room. If the infant did not respond to initial stimulation, umbilical cord milking (UCM) 3–5 times towards the umbilicus was recommended to expedite placental transfusion, or as a substitute for DCC. Preheated linen and an overhead warmer protected the infant from hypothermia. Ventilation support was provided with a T-piece resuscitator (NeoPuff Infant Resuscitator™, Fisher & Paykel, Auckland, NZ). Suction equipment (AGA MS-33 Suction Ejector^TM^, AGA – Linde Healthcare, Oslo, Norway) was applied if necessary. Ventilation support was initiated with room air, controlled by a gas flowmeter (Low-flow Air-Oxygen Blender™, Ohio Medical Corporation, Gurnee, US). Oxygen fraction was increased according to protocol. Pulse oximetry (Nellcor™ OxiMax N-65, Covidien, Boulder CO, US) was recommended. Once stabilized, infants were placed skin-to-skin on the mother’s chest and covered.

### New delivery-room protocol (Period 2)

Preparations, initial assessment and care were provided as described for period 1. In addition, a mobile resuscitation trolley, LifeStart™ (Inspiration Health Care Ltd. Leicestershire, UK) was placed next to the birthing bed. The trolley was tubed to central supplies of air and oxygen, and fully equipped with a T-piece resuscitator, Oxi-blender and suction equipment to facilitate bed-side transitional support and resuscitation. A heating mattress, Cosy-Therm™ (Inspiration Health Care Ltd. Leicestershire, UK) was used for prevention of hypothermia.

**Fig. 1:**
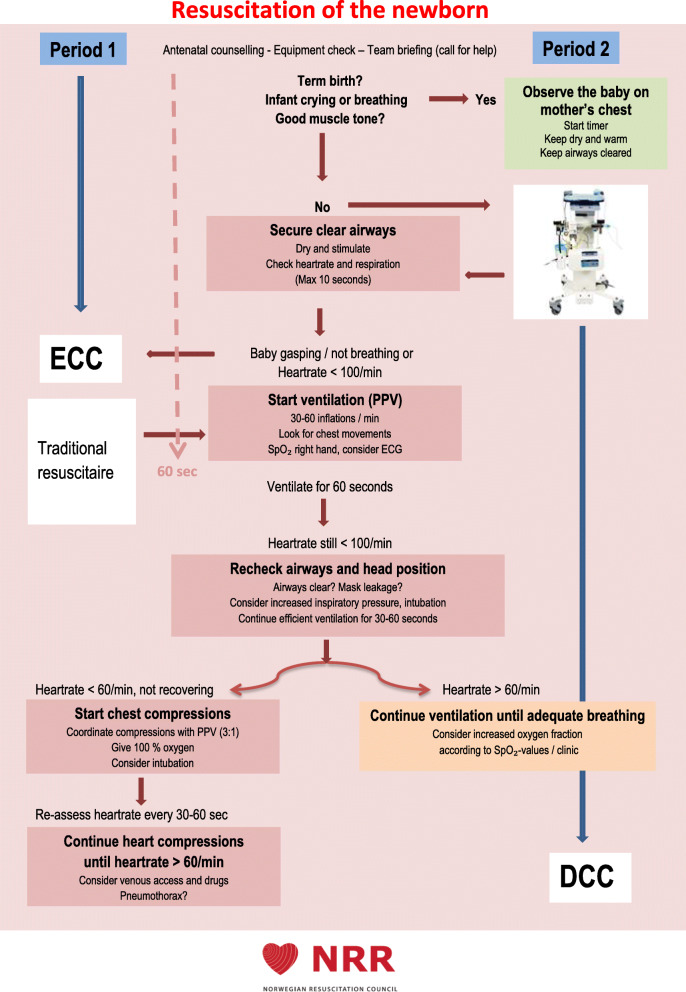
Resuscitation of the newborn. The study interventions in relation to the applied algorithm for resuscitation of the newborn, as recommended by The Norwegian Resuscitation Council (NRR), printed with permission. Photo of the LifeStart™ trolley printed with permission from Inspiration Health Care Ltd

Equipment for assisted vaginal delivery was set up on the opposite side of the birthing bed to avoid logistic problems. If non-vigorous immediately after birth, infants were placed on LifeStart™ for drying, stimulation and further assessment. The algorithm for resuscitation of the newborn by the Norwegian Resuscitation Council (NRR) was applied [33], and modified to include transitional support with intact umbilical cord (Fig. 1). UCM was recommended as described for Period 1. Any ventilation support or resuscitation was initiated with intact umbilical cord. Surveillance by pulse oximetry was recommended. Self-breathing and crying infants were placed directly on mothers’ chest. If the umbilical cord was very short, the trolley was placed as close to the mother’s vagina as possible, otherwise the trolley was placed perpendicular to the birthing bed.

The umbilical cord was cut no earlier than one minute after delivery of the infant; preferably after cease of pulsations or when the cord had turned white. Cord clamping time was recorded. In the event of obstetric emergency requiring better access to the mother, plans were made for how to move the paediatric team quickly. Once stabilized and self-breathing, the infants were placed skin-to-skin with their mothers and covered.

### Inclusion

Live-born singletons with gestational age (GA) ranging from 36 + 0 to 43 + 0 weeks, subject to assisted vaginal delivery (ventouse, forceps or breech), born in the delivery unit during periods 1 and 2 were eligible for inclusion in the QI-project. Vaginal twin deliveries and any converts to emergency caesarean section were excluded.

### Data collection

Data was collected for all consecutive births meeting inclusion criteria. Electronic birth records lacked data on cord clamping; hence data was collected manually for the QI-project. A paper-based form was already routinely in use; collecting data like exact time of birth, birthweight, Apgar scores; serving as support for the subsequent electronic documentation. It was modified to include data on parity, gestational age, delivery mode, indications for assisted delivery and intact cord blood gases. In cases of ECC, attending midwives were asked to fill in indication and whether the cord was milked or not.

For the QI-project, relevant data was registered in an Excel spread-sheet. Once registered, forms were shredded. Data from all deliveries meeting inclusion criteria was extracted from the QI-project data-file and prepared for this study by the QI-project leader. Data on adverse events were extracted from the hospital’s incident reporting system.

### Primary outcome

Prevalence of early cord clamping (ECC)

### Secondary outcomes

- Apgar score at 1,5 and 10 min after birth
- Placement of the infant the first minute after birth (resuscitation table or mother’s chest
- Immediate care of the infant (stimulation/drying, heat-loss prevention, pulse-oximetry)
- Rectal temperature at 10–20 min
- Need for ventilation support
- Need for full resuscitation (chest compressions and positive pressure inflation)
- Transfer to NICU (birth-related reasons).
- Adverse events

### Study of the intervention

The Model for Improvement by Langley [34] was applied in both study periods, representing a theoretical and practical framework for the QI-project. The model’s major tenets are: Setting time-specific and measureable aims, establishing measures to determine if a specific change leads to an improvement, selecting changes that will result in improvement and testing the changes by conducting Plan-Do-Study-Act (PDSA) cycles in a real work setting.

After baseline testing of adherence to cord clamping protocol, an operational target of maximum 2% ECC in vaginal deliveries by November 1st, 2017 was set by the QI-project team. Starting in March, 2017, several PDSA-cycles were carried out to support the improvement efforts.

Identification of drivers and obstacles revealed a gap of knowledge about the benefits of DCC and support of transition with an intact umbilical cord. Thus, building a common knowledge base through cross-professional educational sessions was prioritized. Repeated 1-h standardized presentations of the theoretical rationale was directed by the QI-project team (midwife, neonatologist and NICU-nurse). Cross-professional participation was encouraged to broaden perspectives in plenum discussions. Clinical and practical questions were welcomed and addressed.

Prior to implementation of the new protocol and equipment, mandatory training for all involved personnel was seen as essential to reduce the chances of technical and operational failure or adverse events at the set-out. Training included 1-h theoretical and practical demonstration of set-up and operation of LifeStart™ and associated resuscitation equipment, followed by 2-h simulation training in situ. Simulation included debriefing focusing on inter-disciplinary collaboration and communication. During this preparation, the new delivery room protocol was adjusted according to continuous feedback from discussions and inter-disciplinary hearings.

After implementing the new protocol and equipment, re-education to all involved personnel was offered at several occasions, including brief lectures in the delivery unit and NICU, posters and short simulation sessions. Continuous feedback was welcomed, in order to rule out deviations such as procedural misunderstandings, user errors and technical difficulties. The protocol was again modified, and involved personnel updated, accordingly.

To establish whether the observed outcomes were due to implementation of the new protocol and equipment, or were already detectable before this, variation in cord clamping time was monitored during both study periods, using Statistical Process Control (SPC) analyses [35]. Run charts were used to demonstrate signals of improvement, and whether changes lead to improvement over time. Performance was displayed on an Improvement Board in the delivery unit, for transparency and motivation.

### Statistical analyses

IBM SPSS Statistics software, version 23 was applied for all statistical analysis. *P*-values less than 0.05 were considered to indicate statistical significance. Univariate analyses were performed to identify differences in background variables and cord clamping variables between periods 1 and 2. Continuous variables were compared by using the Mann-Whitney U test or Student’s t-test as appropriate. Categorical / dichotomous variables were compared by using Pearson’s Chi-square (x^2^) test or Fischer’s exact test as appropriate.

Multivariate logistic regression analysis was used to assess the impact of the new delivery room protocol on the prevalence of early cord clamping, adjusted for possible confounding factors. Unadjusted and adjusted odds ratios (OR) were presented with 95% confidence intervals (CI).

The Run Chart was created using Microsoft Excel and analysed using the median and standard run chart evaluation rules. According to the user manual [36], a “shift” represents a period where six or more consecutive points all fall above or below the median, whereas a “trend” occurs when five or more consecutive points all rise or fall.

## Results

**Fig. 2:**
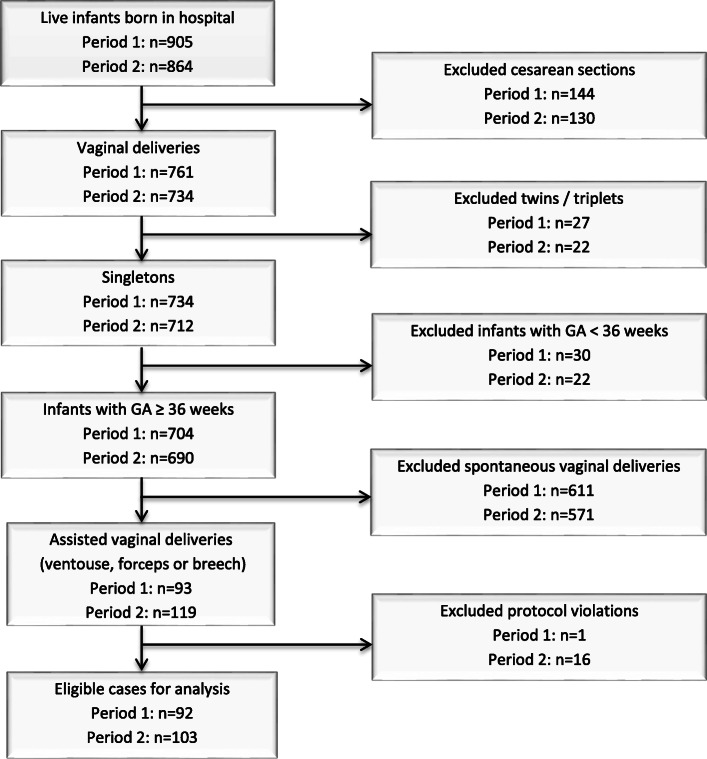
Flow-chart of inclusion of study population

During the QI-project, 905 live infants were born in period 1, compared to 864 in period 2. Of these, 93 were singletons with GA ≥ 36 completed weeks and subject to assisted vaginal delivery in period 1, versus 119 in period 2. A flow chart of inclusion is shown in Fig. 2. A closer investigation of the data revealed 17 protocol violations (assigned protocol not applied). In period 1, this was a case where LifeStart™ was applied before the agreed start-up date. In period 2, violations included: No time for set-up (3), paediatrician declined (9), LifeStart™ not available (3), suction equipment failure (1). All these cases were excluded from statistical analysis.

A total of 195 cases were eligible for statistical analyses in periods 1 (*n* = 92) and 2 (*n* = 103). The characteristics of these are shown in Table 1.

**Table 1:** Background characteristics of mothers and infants

	Period 1 (*n* = 92)	Period 2 (*n* = 103)	*P*-value
Nulliparous	66 (71.7)	74 (71.8)	0.99^a^
Gestational age (weeks)	40.1 ± 1.3	39.9 ± 1.3	0.21^b^
Birthweight (g)	3676 ± 570	3508 ± 437	< 0.01^c^
Delivery mode			
Ventouse	85 (92.4)	94 (91.3)	0.77^a^
Forceps	2 (2.2)	2 (1.9)	1,00^b^
Breech (Lövset’s manoeuvre)	5 (5.4)	7 (6.8)	0.69^a^
Indications, assisted delivery			
Suspect asphyxia^e^	50 (54.3)	61 (59.2)	0.49^a^
Maternal^f^	37 (40.2)	36 (35.0)	0.45^a^
Breech presentation	5 (5.4)	7 (6.8)	0.69^a^
Cord blood gases, artery	73 (79.3)	83 (80.6)	
Arterial pH	7.20 ± 0.1	7.20 ± 0.1	0.81^c^
Arterial PCO_2_ (kPa)	7.34 ± 1.4	7.30 ± 1.3	0.86^c^
Arterial base deficit (mmol/l)	5.93 ± 2.9	5.84 ± 2.9	0.84^c^
Cord blood gases, vein	82 (89.1)	98 (95.1)	
Venous pH	7.32 ± 0.1	7.33 ± 0.1	0.64^c^
Venous PCO_2_ (kPa)	5.08 ± 1.0	5.05 ± 1.0	0.80^c^
Venous base deficit (mmol/l)	5.72 ± 2.5	5.47 ± 2.5	0.53^c^
Metabolic acidosis^g^	0 (0)	1 (0.9)	1.00^d^
Apgar scores			
1 min	8 (6.25–9)	8 (7–9)	0.89^b^
5 min	9 (9–10)	9 (9–10)	1.00^b^
10 min	10 (9–10)	10 (9–10)	0.73^b^

The results are reported as frequency (percentage), mean ± standard deviation (SD), median (inter-quartile range (IQR))

^a^Calculated using Pearson’s Chi-square Test ^b^Calculated using Independent-Samples Mann-Whitney U Test

^c^Calculated using Independent samples T-test, equal variances assumed. ^d^Calculated using Fisher’s Exact Test

^e^Based on information from continuous foetal monitoring (CTG alone or CTG with ST-analysis)

^f^Prolonged 2nd stage, maternal exhaustion, hypertension / pre-eclampsia

^g^Defined as arterial pH < 7.00 and arterial base deficit ≥12 mmol/liter

There were no significant differences in most background variables, except from mean birthweight. At this institution, the preferred method for instrumentation in assisted vaginal deliveries is ventouse. None of the breech deliveries needed instrumentation by forceps. Cord blood analyses were not complete or successful for all cases.

After implementing the new protocol, there was a significant improvement in all cord clamping variables (Table 2). Mean cord clamping time increased by 43%. The main indication for ECC was the need to move infants to a resuscitation table for further assessment and care. This indication did not apply in period 2.

**Table 2:** Comparison of cord clamping variables and indications for ECC

	Period 1 (*n* = 92)	Period 2 (*n* = 103)	*P*-value
Cord clamping time in seconds	320 ± 243	457 ± 375	< 0.001^a^
< 60 s (ECC)	12 (13.0)	1 (1.0)	< 0.01^b^
60–179 s (IMCC)	12 (13.0)	3 (2.9)	< 0.01^b^
≥ 180 s (DCC)	68 (73.9)	99 (96.1)	< 0.001^b^
Umbilical cord milking (UCM)	9 (9.8)	4 (3.8)	0.10^b^
Indications for ECC	(*n* = 12)	(n = 1)	
None / tradition	1 (1.1)	0 (0)	0.47^c^
Infant moved to resuscitation table^d^	11 (12.0)	0 (0)	< 0.001^c^
Cord problems	0 (0)	1 (1.0)	1.00 ^c^
Maternal complications	0 (0)	0 (0)	

The results are reported as mean ± standard deviation (SD), frequency (percentage)

^a^Calculated using Independent samples T-test, equal variances not assumed

^b^Calculated using Pearson’s Chi-square Test ^c^Calculated using Fisher’s Exact test

^d^traditional resuscitation table in period 1, LifeStart in period 2

The only case of ECC in period 2 was due to cord snapping when positioning the infant for transitional support. Ventilation support was given on LifeStart™. The infant was transferred to NICU due to respiration problems and Apgar scores 5–8-8, and the case was reported as an adverse event. The other adverse event was a case of converting care from LifeStart™ to traditional resuscitation table because of a tight cord knot. The cord was clamped at 60 s to move the infant for ventilation support, despite 1 min Apgar score of 9. The cases were investigated in order to avoid future cases, and information on proper care strategies were provided to relevant personnel.

### Variation in prevalence of ECC during the QI-project

**Fig. 3:**
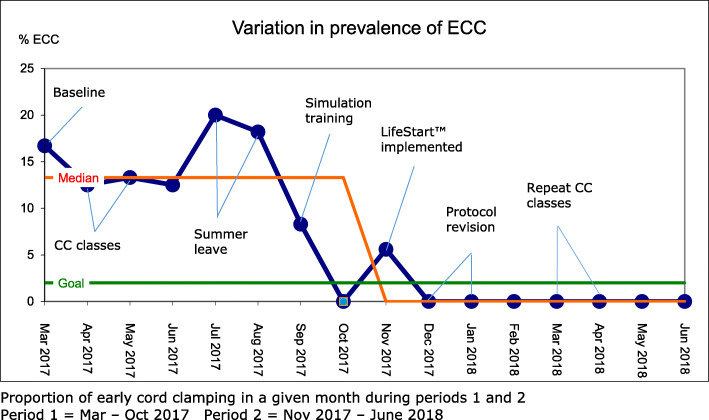
Variation in prevalence of ECC

A Run Chart visualises the variation in prevalence of ECC for periods 1 and 2 (Fig. 3). The blue line reflects proportions of ECC from baseline measurement, through different QI activities to prepare for implementation of the new protocol, to completed implementation.

During simulation training, the prevalence dropped to 8.3% and further to zero, already before implementing the new protocol in November 2018. After protocol revision in December 2018, there were no cases of ECC registered. This fulfills the Run chart requirements of a “shift”, indicating that the improvement was not the result of chance.

### Primary outcome

Multivariate logistic regression was performed to assess the overall impact of the new delivery room protocol on the primary outcome (prevalence of ECC) when adjusted for possible confounders and mediators (Table 3). The proportion of ECC was reduced by 94% in period 2, compared with period 1 (OR = 0.06, 95% CI 0.01–0.49, *p* < 0.01). The only covariate reaching statistical significance was 1-min Apgar score ≤ 5 (*p* < 0.001) When adjusted for, this did not alter the impact of the new delivery room protocol.

**Table 3:** Logistic regression analyses of overall impact of the new delivery room protocol on the prevalence of ECC, adjusted for possible confounding factors

Covariate	Unadjusted OR	95% CI	Adjusted OR	95% CI	*P*-value
Old protocol	Ref.				
New protocol	0.07	0.01–0.51	0.06	0.01–0.49	< 0.01
Nulliparous	0.88	0.26–2.97	1.54	0.29–8.19	0.62
Gestational age	1.19	0.75–1.87	1.08	0.56–2.09	0.81
Birthweight (g)	1.00^a^	1.00–1.00	1.000	1.00–1.00	0.76
Suspect asphyxia	0.88	0.28–2.71	0.84	0.21–3.37	0.80
1-min Apgar ≤5	13.73	4.08–46.22	17.61	4.46–69.50	< 0.001

Analysed for all assisted vaginal deliveries in periods 1 and 2 (*N* = 195)

^a^differences only visible with 3 decimals

*OR* Odds ratio *CI* Confidence interval

After controlling for infants placed directly on mother’s chest after delivery, multivariate logistic regression was performed to assess the direct impact of the new, mobile resuscitation table (Table 4). OR’s were adjusted for the same confounding factors. The likelihood of ECC for infants needing help on a resuscitation table was reduced by 98% when using LifeStart™, compared to a traditional resuscitation Table (OR = 0.02, 95% CI 0.00–0.16, p < 0.001). No covariates reached statistical significance.

**Table 4:** Logistic regression analyses of the direct impact of the new, mobile resuscitation table on the prevalence of ECC

Covariate	Unadjusted OR	95% CI	Adjusted OR	95% CI	*P*-value
Old protocol	Ref.				
New protocol	0.02	0.00–0.13	0.02	0.00–0.16	< 0.001
Nulliparous	0.84	0.23–3.05	0.44	0.06–3.30	0.42
Gestational age	1.22	0.74–2.01	0.95	0.46–1.96	0.89
Birthweight (g)	1.001^a^	1.000–1.002	1.001^a^	0.998–1.003	0.57
Suspect asphyxia	0.73	0.22–2.41	0.63	0.12–3.25	0.58
1-min Apgar ≤5	5.40	1.55–18.83	2.19	0.42–11.39	0.35

Analysed for all infants placed on a resuscitation table in periods 1 and 2 (*N* = 83)

^a^differences only visible with 3 decimals

*OR* Odds ratio, *CI* Confidence interval

### Secondary outcomes

All infants were initially stimulated by drying and rubbing their back with preheated towels. Heat-loss prevention was also provided for all cases; in period 1 by an overhead warmer; in period 2 by a heating mattress. 20% of the infants in period 2 had pulse-oximetry attached, compared to zero in period 1. ECG-monitors were not available throughout the project. No infants needed full resuscitation in either period. Univariate analysis showed that infants in period 2 were significantly less likely to be placed on mothers’ chest (within the first minute of life), despite better 1-min Apgar scores. However, they were less likely to receive ventilation support or to be transferred to NICU (Table 5).

**Table 5:** Comparison of immediate transitional care and short-term outcomes for infants placed on a resuscitation table^c^ in periods 1 and 2

Place of transitional care	Period 1 (*n* = 92)	Period 2 (*n* = 103)	*P*-values
Infants placed on mothers chest	69 (75.0)	43 (41.7)	< 0.001^a^
Infants placed on resuscitation table	23 (25.0)	60 (58.3)	< 0.001^a^
Short-term outcomes for infants placed on a resuscitation table^c^	(*n* = 23)	(*n* = 60)	
Apgar 1 < 5	12 (52.0)	12 (20.0)	< 0.01^a^
Apgar 5 < 7	4 (17.4)	3 (5.0)	0.09^b^
Apgar 10 < 9	9 (39.0)	9 (15.0)	0.05^a^
Ventilation support	15 (65.2)	33 (55.0)	0.40^a^
Transfer to NICU	6 (26.1)	7 (11.7)	0.17^a^

The results are reported as frequency (percentage)

^a^Calculated using Pearson’s Chi-square Test ^b^Calculated using Fisher’s Exact Test

^c^traditional resuscitation table in period 1, LifeStart in period 2

Infants in period 2 were less likely to have 5-min Apgar < 7 or 10-min Apgar< 9, but after adjusting for possible confounding factors (parity, GA, birthweight, suspect asphyxia and low 1-min Apgar score) by multivariate logistic regression analysis, no significant difference could be demonstrated for any of the short-term outcomes (Table 6). Pre-existing indications (maternal diabetes) and late-onset complications (infection, heart conditions, drugs) resulting in subsequent interventions on a resuscitation table or transfer to NICU were excluded from analysis, since this did not interfere with cord clamping practice.

**Table 6:** Logistic regression analyses of immediate care and short-time outcomes for infants placed on a resuscitation table^a^ in periods 1 (n = 23) and 2 (n = 60)

	Unadjusted estimate			Adjusted estimate		
	OR	95% CI	*P*-value	OR	95% CI	*P*-value
Old protocol	Ref.					
Ventilation support	0.65	0.24–1.77	0.40	1.22	0.38–3.97	0.74
Low Apgar scores						
Apgar 5 < 7	0.25	0.05–1.22	0.08	0.79	0.10–6.37	0.82
Apgar 10 < 9	0.27	0.09–0.82	0.02	0.57	0.15–2.17	0.41
Transfer to NICU^b^	0.37	0.11–1.27	0.11	0.67	0.16–2.76	0.58

Adjusted for parity, gestational age, birthweight, suspect asphyxia and 1-min Apgar score ≤ 5

^a^traditional resuscitation table in period 1 (*n* = 23), LifeStart in period 2 (*n* = 60)

^b^due to prolonged ventilation support or unsuspected compromised infant at birth

*OR* Odds ratio, *CI* Confidence interval

## Discussion

This QI cohort study demonstrates that implementing a new delivery room protocol involving a mobile resuscitation trolley (LifeStart™) successfully eliminates early cord clamping in assisted vaginal deliveries. The systematic approach to practice change provided by the Model for Improvement, seems crucial for both achieving as well as sustaining the targeted results.

The strongest effect on the prevalence of ECC was seen for infants actually placed on a resuscitation table within the first minute after birth, with a relative risk reduction of 98% when using LifeStart™ compared to traditional equipment. At the same time, we saw an unintended and unexpected effect of the new protocol: infants were less likely to be placed directly on mother’s chest after introduction of LifeStart™. This included vigorous, as well as non-vigorous infants. Other studies involving mobile resuscitation equipment have not reported this [22, 37]. Several explanations may be plausible: It may reflect reactivity to the study situation, or the researcher’s expectations, given that all personnel had been trained by the researcher in operating the new equipment, and was aware of being monitored with regards to protocol adherence. It may reflect novelty effects, where enthusiastic personnel finally had the opportunity to use the new equipment in real life situations. A more pragmatic explanation; given the mothers’ legs placed in stirrups for delivery; may be that the mobile trolley was a convenient station to place infant when sampling for cord blood gases. Although direct skin-to-skin contact between mother and baby was interrupted, the parents could still watch, touch and comfort their baby during transition, as valued positive in the study by Sawyer et al. [38]. An increased focus on incorporating Kangaroo Care [39] in these situations would probably have reduced separation.

In line with recent feasibility studies [22, 37], no infants were compromised enough to require full resuscitation. Many infants were stabilized after drying and stimulation only. Once on the LifeStart™, the infants’ placement was probably quite transient; since around 45% did not receive ventilation support. Rather reassuringly, infants cared for on LifeStart™ were neither more likely to have low 5- and 10-min Apgar scores nor being transferred to the NICU for respiration problems, compared with infants placed on a traditional resuscitation table.

In this study, the proportion of infants with DCC (> 180 s) increased from 74 to 96%, and the mean cord clamping time increased from 5 min 20 s to 7 min 37 s after implementation of the new delivery room protocol. This is comparable to the results for normal vaginal deliveries during the QI-project. It exceeds the recommendations in a recent RCT including term infants, showing that a delay in cord clamping of at least 5 min results in higher iron levels, and may be beneficial for the infant’s brain development due to higher myelin content [21]. It is also comparable to the mean cord clamping time found in a feasibility-RCT on term infants at risk for resuscitation [22], where infants randomized to more than five minutes delay displayed better central blood pressure and oxygen saturation of the brain.

Other researchers have suggested that using a stopwatch to time cord clamping may be inappropriate, especially in situations where infants are at risk for resuscitation. They conclude that timing should rather be based on the infant’s physiology, more specifically if the infant is breathing or not [15, 18, 37]. In our study, postponing the decision to clamp the cord allowed the obstetric and paediatric teams to concentrate on the immediate medical needs of infant and mother. Timing of cord clamping was left to the discretion of midwives, whose approach for centuries has been after cease of pulsations or after delivery of the placenta.

To our knowledge, this is the first QI-study to evaluate implementation of transitional support with an intact umbilical cord in assisted vaginal deliveries specifically. Other QI-studies have focused on implementing or expanding protocols of delayed cord clamping for premature infants; reporting on the inverse outcome (prevalence of delayed cord clamping); with operational targets of 80%. Achieved results vary between 53.5 and 85% [40, 41]. The current study; reporting on early cord clamping; is not directly comparable due to differences in baseline and background variables. Nevertheless, the operational target (2% ECC) is reached faster; and seems more successfully sustained over time; as only one case of ECC was registered after implementation of LifeStart™. However, a pattern of improvement was detectable already before the implementation of the new protocol, most markedly following mandatory “cord clamping classes” and interdisciplinary simulation training; suggesting that increased focus alone may induce practice change. The marked increase in proportion of ECC during summer leave may reflect that 30–40% of ordinary personnel were replaced by substitutes; not necessarily well acquainted with the protocol. This underpins the necessity of continual training and education rounds to reach new employees, as highlighted in previous QI-studies [40, 41].

Other approaches to intact-cord stabilization have also been proven feasible. Batey and colleagues [42] describe how intact-cord stabilization of high-risk infants can be done either with a standard resuscitation table or the LifeStart™ trolley. The Baby-DUCC feasibility study [37] is another example of how this may be done. However, feedback from involved personnel in our study revealed that behind the seemingly simple intervention of moving equipment and personnel to the potentially compromised infant, lay several logistic challenges, as well as communication and collaboration issues. These issues have also been encountered by other researchers [29, 37, 43, 44]. Examples include: ensuring availability and readiness of the equipment with several deliveries going on at the same time, collaboration and planning ahead in rapidly progressing deliveries, and how to manage space constraints at the birthing bed. Applying the Model for Improvement [34] to these challenges not only ensured a systematic approach, but also constituted a way of addressing the root causes of undesired variation.

### Safety issues

This study demonstrates that transitional care and ventilation support can be done with an intact umbilical cord in assisted vaginal deliveries by moving personnel and equipment to the infant. However, the potential problem of overstretching or kinking the cord, represented by the one case of cord snapping when using mobile equipment, is a safety issue that needs to be addressed. Other researchers have also described this challenge, and how it can be overcome, for example by placing the trolley closest possible to the birth canal [37, 42].

This study implied the use of new technology under potentially urgent and stressful conditions. Considerable effort was made to ensure that all involved personnel expected to operate the trolley and associated resuscitation equipment, were trained for and confident in its use. For the project period and beyond, a standard resuscitation table was present and ready in the delivery-room in case of procedural failure. If obstetric or paediatric team was not confident about the safety of the mother or infant, they could convert to traditional care. Resuscitating an infant at the mother’s bedside may also impose considerable stress on parents as well as the involved personnel. However, feedback from parents and care-providers in feasibility studies has been predominantly positive [38, 44]. For the actual setting, the major change was performing face-to-face and being able to communicate with the parents, instead of turning backs on them in the opposite end of the room. In other settings, the alternative to bed-side care may be providing care in another room, constituting a greater change for the involved personnel and possibly requiring more time to adjust.

### Strengths and limitations

Choosing a QI-design for the current study was primarily done due to availability of data and funding. It may be seen as a limitation that the study lacks data on outcome parameters like infant haemoglobin, bilirubin and haematocrit, or transitional data like saturation, heartrate and expired CO2. It was not set up or powered to detect changes in morbidity or mortality, and data on maternal outcomes like postpartum haemorrhage or placenta problems were incomplete. However, numerous randomized controlled trials have already demonstrated the benefits of DCC and the potentially harmful effects of ECC [8, 21]. Recent studies have supported the findings, both for vigorous and non-vigorous infants [15, 22, 26]. This study translates the findings from previous research into clinical practice.

Analyses were done as per protocol. However, excluding protocol violations from the analyses may represent some selection bias. 16 of 17 violations happened in period 2, either because the mobile trolley was not available or set up and ready for use, or due to paediatric teams wanting to use traditional equipment instead. Three cases (one in each category) resulted in ECC. From a QI perspective; to assess whether the implementation of the new protocol was successful, these cases should perhaps have been included in the Run Chart, but they are not.

The delivery indication “suspect asphyxia” represents a known risk factor for ECC, by being closely associated with need for support on a resuscitation table. In this study, the term “suspect asphyxia” refers to a situation preceding the intervention, thereby constituting a possible confounder. Delivery room protocols, complications in 2nd and 3rd stage of labour and low 1-min Apgar scores were thought to represent potential mediators. In clinical situations, “born non-vigorous” is normally diagnosed within 10–15 s of life according to Neonatal Resuscitation Guidelines [11]. The decision to move the infant to a resuscitation table is frequently made within the first 30–60 s. The on-going and continuous judgement of the infant’s condition during the 2nd stage and upon complete delivery of the infant, culminating at actual assessment of the 1-min Apgar score, may be seen as a mediating factor influencing on caregivers’ decision to clamp the cord. Second and 3rd stage complications like shoulder dystocia, cord related problems and unexpected non-vigorous infant are closely related to poor 1-min Apgar scores and may therefore represent a problem of multi-collinearity in regression analyses. Hence, data on 1-min Apgar was used as a proxy for infant complications in 2nd and 3rd stage of labour in the statistical analyses.

Health care providers’ experience and confidence in neonatal transitional care, collaboration and communication between and within the obstetric and paediatric teams, and available time for planning and preparations prior to delivery may also represent confounding factors. This study did not specifically collect data on these factors, but their possible contributions are partly accounted for in the Statistical Process Control-analysis.

Upon introducing the mobile resuscitation trolley, some members of the paediatric teams raised concern about how to prevent infant hypothermia, since the trolley’s only source of prevention was a heating-mattress. Pilot testing of the trolley had not reported higher rates of hypothermia for term infants [29]. For the current study, data on rectal temperature within 15 min after birth was initially included in the data collection plan. However, going through data from both cohorts revealed that this information was lacking in too many cases, making analysis meaningless. This is a clear limitation for analysis of short-term outcomes. On the other hand, as mentioned in the Baby-DUCC feasibility study [37], it can be argued that term and near-term infants are not the most vulnerable for hypothermia, especially not when transfused with warm blood from the placenta and dried and covered by warm towels straight after birth.

Furthermore, the study does not provide any formal evaluation of the new equipment. Prior to implementation of the new delivery room protocol, a simple questionnaire was developed, in order to get clinicians’ views of the new trolley compared with traditional equipment. The questions were based on a questionnaire used in a recent feasibility trial [29]. Oral and written information about the questionnaire and its objectives was spread to all involved personnel. Despite numerous reminders, the response-rate from members of the paediatric teams (who actually operated the trolley in most cases) remained low. Instead of analysing the scarce and possibly biased material, all written feedback was incorporated in the PDSA-circles of the QI-project.

### Implications for the future

Neonatal transitional support with an intact umbilical cord is suggested by WHO, provided that equipment and personnel are available at the mother’s bedside [10]. In non-vigorous babies, establishing effective ventilation often takes longer than the recommended 1 min. ECC puts the infant in an unstable situation where oxygenation may be inadequate and cardiac output compromised. This study demonstrates one way of overcoming this obstacle, whereby the benefits of placental transfusion; including oxygen, iron, nutrients and millions of stem cells are made available for infants needing it the most. The next steps should include expanding the protocol for all gestations and when caesarean sections are indicated, preferably combined with Kangaroo Care [39].

Our study has demonstrated the importance of testing and monitoring adherence to protocol in order to assess whether or not operational targets are reached and observed variation is acceptable. This requires reliable information and documentation. Reliable data is also crucial for research purposes. Currently, the Norwegian Medical Birth Registry does not require documentation on cord clamping. Hence this information is often omitted in birth records. This makes both monitoring quality and research involving cord clamping difficult. Most importantly, the possible confounding effect of different cord clamping practices cannot be controlled or adjusted for in research on short- and long-term birth-related outcomes neither for infants nor for mothers.

## Conclusions

Our study demonstrates that implementing a new delivery room protocol involving mobile resuscitation equipment successfully eliminated early cord clamping in assisted vaginal deliveries. By this approach, full attention could be given to the immediate care of the infant. The decision of cord clamping could be postponed and tailored to meet the physiological needs of the infant instead of being dictated by logistics or time. The Model for Improvement appeared to be a useful tool for monitoring adherence to protocol as well as bringing about and sustaining desired change. Future protocol expansions should preferably include Kangaroo Care to avoid unnecessary separation of mothers and infants. Reliable documentation of umbilical cord clamping may help researchers and health professionals in defining optimal practice for the future.

## Data Availability

The datasets used during the current study are available from the corresponding author on reasonable request.

## Abbreviations

CI: Confidence interval
DCC: Delayed cord clamping
ECC: Early cord clamping
ECG: Electrical cardiograph
GA: Gestational age
ICC: Immediate cord clamping
IMCC: Intermediate cord clamping
NICE: National Institute for Health and Care Excellence
NICU: Neonatal Intensive Care Unit
NRR: Norwegian Resuscitation Council
OR: Odds ratio
PDSA: Plan-Do-Study-Act
PPV: Positive pressure ventilation
QI: Quality improvement
SPC: Statistical Process Control
STAN: ST-Analysis of the foetal cardiograph
UCM: Umbilical cord milking
WHO: World Health Organization
